# Prospects of marine-derived compounds as potential therapeutic agents for glioma

**DOI:** 10.1080/13880209.2024.2359659

**Published:** 2024-06-12

**Authors:** Ying Liu, Zhiyang Zhou, Shusen Sun

**Affiliations:** a Department of Pathology, Xiangya Changde Hospital, Changde, China; b Department of Breast Surgery, Xiangya Hospital, Central South University, Changsha, China; c National Clinical Research Center for Geriatric Disorders, Xiangya Hospital, Central South University, Changsha, China; d College of Pharmacy and Health Sciences, Western New England University, Springfield, MA, USA

**Keywords:** Marine environment, natural products, treatment

## Abstract

**Context:**

Glioma, the most common primary malignant brain tumour, is a grave health concern associated with high morbidity and mortality. Current treatments, while effective to some extent, are often hindered by factors such as the blood–brain barrier and tumour microenvironment. This underscores the pressing need for exploring new pharmacologically active anti-glioma compounds.

**Methods:**

This review synthesizes information from major databases, including Chemical Abstracts, Medicinal and Aromatic Plants Abstracts, ScienceDirect, SciFinder, Google Scholar, Scopus, PubMed, Springer Link and relevant books. Publications were selected without date restrictions, using terms such as ‘*Hymenocrater* spp.,’ ‘phytochemical,’ ‘pharmacological,’ ‘extract,’ ‘essential oil’ and ‘traditional uses.’ General web searches using Google and Yahoo were also performed. Articles related to agriculture, ecology, synthetic work or published in languages other than English or Chinese were excluded.

**Results:**

The marine environment has been identified as a rich source of diverse natural products with potent antitumour properties.

**Conclusions:**

This paper not only provides a comprehensive review of marine-derived compounds but also unveils their potential in treating glioblastoma multiforme (GBM) based on functional classifications. It encapsulates the latest research progress on the regulatory biological functions and mechanisms of these marine substances in GBM, offering invaluable insights for the development of new glioma treatments.

## Introduction

Glioma, especially glioblastoma multiforme (GBM), is the predominant primary intracranial tumour, accounting for approximately 50.1% of all malignant brain tumours (Nguyen et al. 2021). An average annual mortality rate of 4.41 per 100,000 population represents a significant health challenge (Ostrom et al. 2022). The gold standard for GBM treatment involves surgical resection, radiotherapy and chemotherapy (Lang et al. 2021). However, despite these efforts, the primary chemotherapeutic agent for GBM, temozolomide (TMZ), only produces a median overall survival (OS) of 14.6 months (Sun et al. 2021). Several factors contribute to the limited effectiveness of standard therapies. The activation of DNA repair pathways, the presence of the blood–brain barrier (BBB), and the complex tumour microenvironment collectively hinder treatment outcomes, resulting in a bleak prognosis for patients with GBM (Delello Di Filippo et al. 2021; Tomar et al. 2021; Cai et al. 2023). Consequently, the exploration of new, safe chemical compounds exhibiting antitumour pharmacological activity, along with the development of innovative therapeutic approaches, remains crucial. These efforts are essential in the ongoing quest to create more effective drugs capable of significantly improving the survival rates of individuals diagnosed with GBM.

With the advancement of human technology in ocean exploration, an increasing number of marine source materials are being studied in-depth worldwide. The marine environment has become a rich reservoir of various natural products, which exhibit significant biological activities, including cytotoxic, antibacterial, antifungal, antiviral, antiparasitic, antitumour and immunomodulatory properties (Zhou X et al. 2013; Norris and Perkins 2016; Abdelmohsen et al. 2017; Guo K et al. 2021; Bohringer et al. 2023). Approximately 49 marine-derived active substances or their derivatives have been approved or entered clinical trials (Zhang QT et al. 2021). Many of these bioactive molecules are extracted from marine sponges, mollusks and algae. Currently, 11 types of marine drugs have been approved for clinical use by European and US Drug Authorities, five demonstrating anticancer efficacy: Cytosar-U, Yondelis, Halaven, Adcetris and Aplidin (Jimenez et al. 2020). Marine-derived compounds have exhibited potent antitumour effects against various cancers, including ovarian, breast, liver, lung and colorectal cancer (Terasaki et al. 2019; van Rixel et al. 2019; Binnewerg et al. 2020; Li W et al. 2020; Zhou B et al. 2021). These compounds exert anticancer activities through multiple mechanisms, such as inhibiting tumour growth, impeding cancer cell migration, inducing apoptosis and causing cell cycle arrest (Liberio et al. 2014; Oliveira et al. 2019; Piazzini et al. 2019; Xie Q et al. 2019). Several *in vitro* experiments have demonstrated the inhibitory effects of different doses of marine-derived organic compounds on the biological functions of glioma cells, offering promising avenues for treating GBM (Alves et al. 2021; Khotimchenko et al. 2021). However, despite the demonstrated potential of many marine-derived substances in GBM, none has yet been approved as a drug specifically for the treatment of this formidable brain cancer.

**Figure 1.**
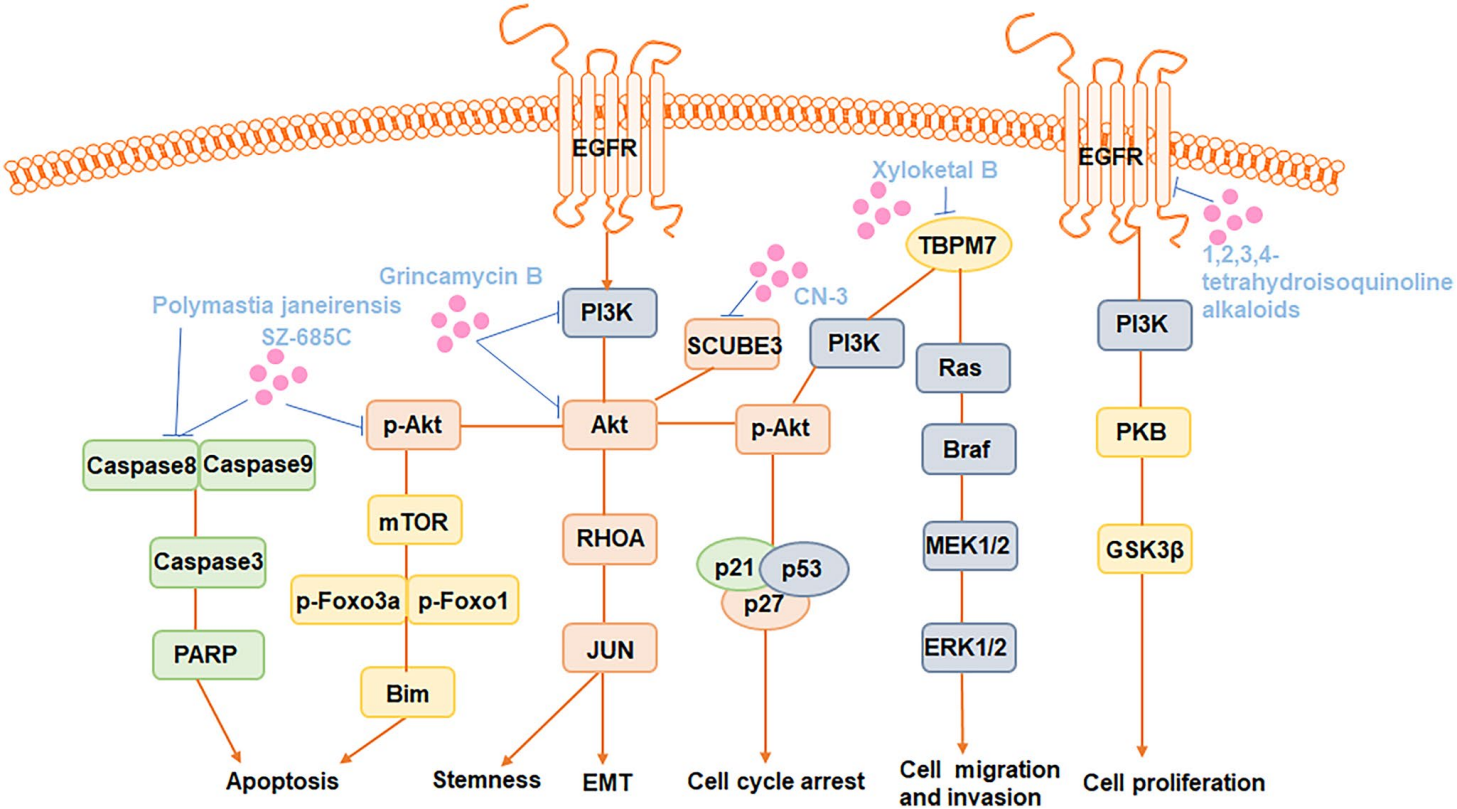
Major targeted pathways regulated by the marine-derived compounds in the glioma cell.

Numerous studies have highlighted the inhibitory effects of marine-origin compounds and their derivatives on glioma cell growth, apoptosis induction, autophagy stimulation and cell cycle arrest (Jarry et al. 2014). Abnormalities in multiple signalling pathways, including the epidermal growth factor receptor (EGFR) signalling pathway, the phosphatidylinositol 3-kinase (PI3K)/protein kinase B (AKT)/mammalian target of rapamycin (mTOR) pathway and the extracellular signal-regulated kinase (ERK)/mitogen-activated protein kinase (MAPK) signalling pathways, are involved in the anti-glioma effects of marine-derived natural products (Figure 1).

Among them, the PI3K/AKT/mTOR pathway is the crucial pathway highly involved in cell proliferation, growth, migration and cell cycle arrest in glioma. The interaction of EGFR with tyrosine kinase activates PI3K, leading to the phosphorylation of phosphatidylinositol 4,5-bisphosphate (PIP2) and its conversion into phosphatidylinositol 3,4,5-trisphosphate (PIP3). PIP3 activation reduces AKT activation while increasing the expression of mTOR complexes 1 and 2 (Ramar et al. 2023). Activation of mTORC1 increases cellular development by enhancing the anabolic process of other macromolecule formation while decreasing the catabolic response. In contrast, activation of mTOR complex 2 improves cell survival and proliferation (Obrador et al. 2024). The Ras/RAF/MEK/ERK pathway is hyperactive in glioma cells (Schreck et al. 2019). Activation of the Ras protein occurs through the replacement of GDP by GTP, initiating the activation of the MAP kinases. These kinases phosphorylate and activate downstream ERK proteins, which promote glioma cell proliferation and migration (Obrador et al. 2024). For example, Xyloketal B, a novel marine compound isolated from the mangrove fungus *Xylaria* sp. (No. 2508), exerts its effects by overexpressing the TRPM7 and suppressing the PI3K/Akt and MEK/ERK signalling pathways, thus contributing to antiproliferation and migration effects in GBM (Chen WL et al. 2015).

Marine-derived natural compounds have higher toxicity against GBM than TMZ, making them promising candidates for chemotherapy (Alves et al. 2021; Peng et al. 2023). However, it is essential to recognize that research on marine-derived compounds is still in the early stages of drug development. Most of their bioactivity is evaluated based on the half-maximal inhibitory concentration (IC_50_) of the cells.

**Figure 2.**
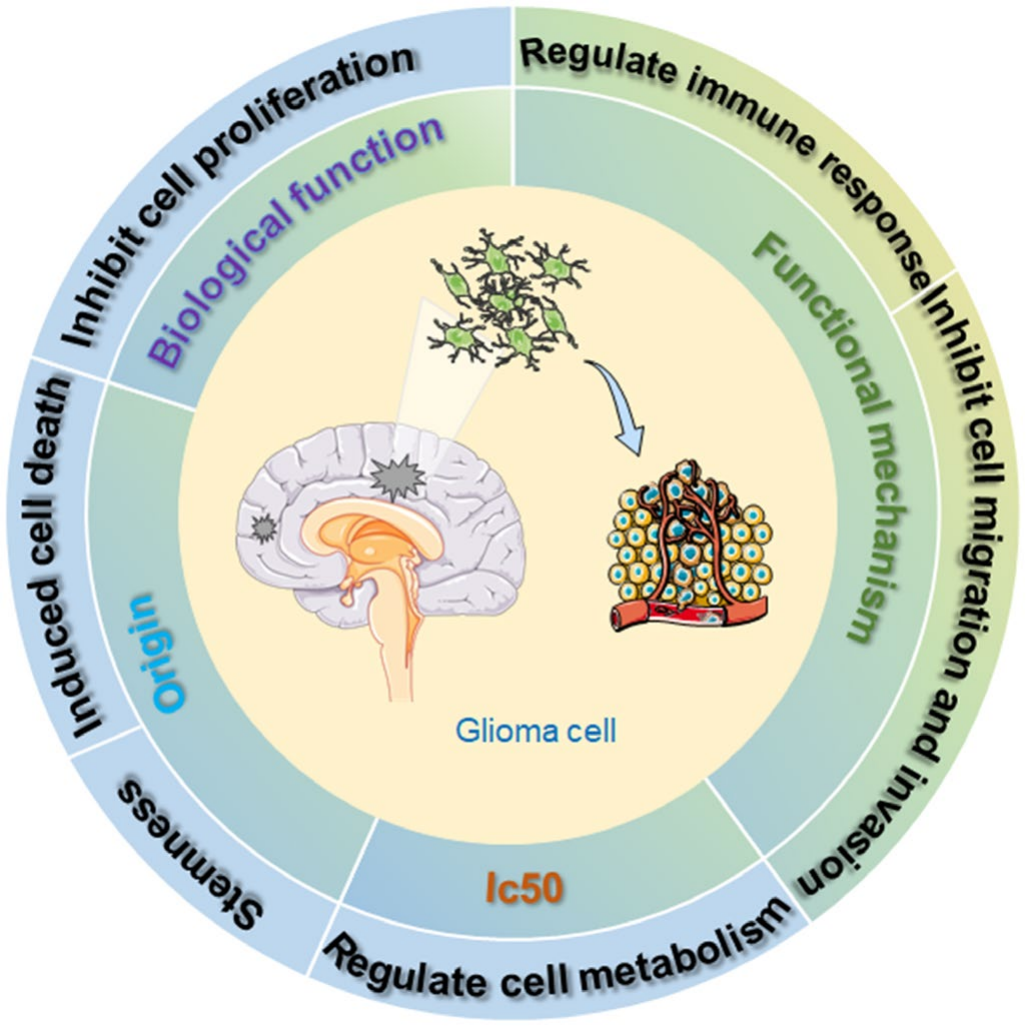
Marine-derived compounds affect the biological activity of glioma cells.

This review systematically and comprehensively examines the effects of marine-sourced materials on GBM, discussing their advantages and disadvantages based on existing research findings. This analysis provides a valuable reference for future research. It details the physiological impacts of marine-derived substances on glioma over the past two decades, highlighting their roles in inhibiting cell proliferation, inducing apoptosis and autophagy, and triggering cell cycle arrest (Figure 2). Subsequent sections elaborate on these biological functions, providing practical insights into potential therapeutic applications.

## The marine-derived natural products that affect the proliferation of glioma cells

In various pathological conditions, specific agents, such as environmental and chemical factors, have the potential to initiate the carcinogenesis process by inducing successive rounds of cell proliferation and clonal selection (Kasprzak 2023). Numerous natural compounds exhibit biological activities that inhibit cancer cell proliferation (Mohammadi et al. 2022), making them viable candidates for chemotherapeutic strategies.

### Natural anti-glioma products from marine-associated actinomycete Streptomyces

The primary biological function of effective anticancer compounds lies in their ability to hinder tumour cell viability and impact cell proliferation. Given the diverse marine sources and their derivatives, we discuss these compounds according to their origin. Marine-derived actinomycete *Streptomyces* are crucial reservoirs for discovering novel bioactive natural products. One such extraction was derived from the marine actinomycete *Streptomyces* sp. culture. P11-23B produced the streptodepsipeptides P11A and P11B (**1**). The streptodepsipeptide P11A exhibited significant activity against proliferation, demonstrating inhibitions of 87.17% for U87-MG glioma cells and 86.84% for U251 cells. Both streptodepsipeptides, P11A and P11B, showed potent activity with IC_50_ values ranging from 0.3 to 0.4 μM and 0.1 to 1.4 μM, respectively (Ye et al. 2017).

Marine-derived actinomycetes from the *Streptomycetaceae* family, such as photopiperazines A–D (**2**) (Kim et al. 2019), hygrocins C (**3**) (Yi, Newaz, et al. 2022), actinomycins (**4**) (Zhang X et al. 2016), grincamycin B (**5**) (Yao et al. 2020), marinacarbolines E (**6**) (Qin et al. 2020), exhibited inhibitory activities against glioma cell proliferation. Photopiperazines showed an IC_50_ value of 0.12 μM in U87 cells, while hygrocins C showed the most potent activity against U87MG and U251 glioma cells with IC_50_ values of 0.16 μM and 0.35 μM, respectively. The IC_50_ values for actinomycins in U251, SHG44 and C6 glioma cells range as follows: 1.01–10.06 nM for actinomycin D, 0.42–1.80 nM for actinomycin V and 3.26–25.18 nM for actinomycin X0β. The control drug doxorubicin exhibited activity with IC_50_ values ranging from 0.70 to 9.61 μM. Marinacarbolines E showed cytotoxic activity against U87MG cells with IC_50_ values of 2.3–8.9 μM.

Several bioactive compounds isolated from marine *Streptomyces*, for example, polycyclic quinones (**7**) (Liang et al. 2016), Streptoglutarimides A–J (**8**) (Zhang D et al. 2019) and bafilomycins (**9**) (Zhang X et al. 2017; Zhang Z et al. 2017), streptonaphthyridine A (**10**) (Qin et al. 2023), seco-geldanamycin A (**11**) (Yi, Lian, et al. 2022), bagremycin C (**12**) (Chen L et al. 2017) and *Streptomyces* sp. ZZ406 (**13**) (Chen M et al. 2018) showed potent efficacy in suppressing proliferation of tested glioma cell lines. Anthraquinones exhibited a notable inhibition of proliferation in four different glioma cell lines, with IC_50_ values ranging from 0.5 to 7.3 μM, and induced apoptosis in these glioma cells. ZZ741 streptoglutarimide H and streptovitacin A demonstrated a potent antiproliferative activity against human glioma U87MG and U251 cells, with IC_50_ values ranging from 1.5 to 3.8 μM and 0.05 to 0.22 μM, respectively. The four bafilomycins exhibited potent activity in suppressing the proliferation of U87-MG, U251 and SHG-44 cells and C6 cells from rat glioma, with IC_50_ values ranging from 0.35 to 2.95 µM. The positive control doxorubicin showed similar activity with IC_50_ values of 0.48–1.76 µM. Streptonaphthyridine A suppressed proliferation in U87MG and U251 cells with IC_50_ values of 7.9 ± 1.3 and 13.4 ± 2.7 µM, respectively. Bagremycin C was active against four glioma cell lines, with IC_50_ values ranging from 2.2 to 6.4 μM. The IC_50_ values for ZZ406 in different glioma cells range from 4.7 μM to 8.1 μM. These results underscore the robust activity of these natural product molecules isolated from marine-derived *Streptomyces* in inhibiting glioma cell proliferation.

A cytotoxic peptide, newly isolated from the marine-derived bacterium *Brevibacillus sp*. S-1, demonstrated cytotoxicity against U251 human glioma cells (Zheng et al. 2014). This discovery implies that various strains of marine origin may possess anti-glioma growth effects, opening avenues for further exploration and investigation.

### Natural anti-glioma products from marine-associated fungi

Marine-derived fungi serve as abundant reservoirs for natural products characterized by their uniqueness in structure and bioactivity. They play a pivotal role in exploring new bioactive compounds and identifying potential drug-lead compounds. Fungi belonging to *Penicillium* species have emerged as prolific producers of new bioactive compounds. Penicipyrroether A **(14**) identified from the marine-derived *Penicillium sp. ZZ380* represents a promising chemical compound with antitumour pharmacological activity. This compound exhibits potent antiproliferative effects against human glioma U87MG and U251 cells, with IC_50_ values ranging from 1.64 to 5.50 μM (Song et al. 2019). A culture of the marine-associated fungus *Penicillium sp.* ZZ1750 in rice medium produced seven new compounds, peniresorcinosides A–E, penidifarnesylin A, penipyridinone A and penipyridinone B. Peniresorcinosides A and B (**15**), classified as rare glycosylated alkylresorcinols, demonstrated potent antiglioma activity. They exhibited IC_50_ values of 4.0 and 5.6 µM against U87MG cells and 14.1 and 9.8 µM against U251 cells, respectively (Yong et al. 2021). Penipyridinone B (**16**) had a potent anti-glioma activity, with IC_50_ values of 2.45 μM for U87MG cells and 11.40 μM for U251 cells (Yong et al. 2022).

Beyond *Penicillium* species fungi, various marine fungi compounds have shown cytotoxic effects against glioma. Xyloketal B, a novel marine compound isolated from the mangrove fungus *Xylaria sp*. (No. 2508) in the South China Sea, has been identified as one such compound (Lin et al. 2001). Xyloketal B (**17**) exerts its effects by suppressing the PI3K/Akt and MEK/ERK signalling pathways, contributing to antiproliferation and migration effects in GBM (Chen WL et al. 2015). Its IC_50_ was measured at 287.1 ± 1.0 μM. Terpestacin (**18**), isolated from the fermentation broth of the coral endophytic fungus *Arthrinium* sp. SCSIO41221 has also shown promising effects. Specific compounds (**5**, **11**, **13** and **15**) showed a strong ability to inhibit proliferation (IC_50_ 2.8–6.9 μM) and the formation of tumour spheres of glioma stem cells (GSCs). Compounds **13** and **15** effectively induced apoptosis and significantly inhibited GSC invasion (95% and 97% inhibition, respectively, at 2.5 μM) (Liao et al. 2023).

Gliotoxin (**19**), a hydrophobic fungal metabolite from the ocean, binds directly to pyruvate kinase M2 (PKM2) in the human glioma cell line U87. This interaction reduces glucose metabolism and induces cell death, with an IC_50_ value of 0.54 mM in U87 cells, indicating antitumour activity (Tang W et al. 2020). In summary, numerous compounds extracted from marine fungi inhibit glioma cell proliferation, suggesting their potential for prevention and treatment (Li Q et al. 2018; Zhang D et al. 2021).

### Natural anti-glioma products from marine-associated animals

Extracts from marine animals, including sponges, ascidians, jellyfish and others, have shown significant inhibitory effects on glioma growth and progression, offering a new avenue for treatment. Four decades ago, the isolation of C-nucleoside from a Caribbean sponge laid the foundation for the synthesis of cytarabine. Cytarabine is the first marine-derived anticancer agent developed for clinical use and is currently a routine treatment for leukaemia and lymphoma (Schwartsmann et al. 2001). Sponge metabolites, known for their diverse pharmaceutical potential, exhibit various activities, including anticancer, anti-inflammatory, antiviral and antibacterial properties (http://marinepharmacology.midwestern.edu/clinPipeline.htm). Stellettin B (**20**), isolated from the marine sponge *Jaspis stellifera*, Carter 1879 (Ancorinidae), has shown remarkable inhibition against human glioblastoma cell line SF295 growth, with *in vitro* IC_50_ values of 0.01 μM. Its cytotoxic effects on human cancer cells exhibit relative selectivity compared to normal cell lines. The antiproliferative effects of stellettin B may involve inhibition of the PI3K/Akt pathway.

Stellettin B promotes PI3Kα degradation through the ubiquitination-proteasome pathway, reducing the expression of key components of the homologous recombination (HR) repair pathway, such as BRCA1, BRCA2 and RAD51. This mechanism improves the sensitivity of GBM cells to ionizing radiation by delaying DNA damage repair, ultimately resulting in GBM cell death. Stellettin B is a potential lead compound to discover promising drug candidates in treating certain glioblastomas (Tang SA et al. 2014; Peng et al. 2023).

Calcium channels are common targets for natural toxins, with two notable families of guanidine alkaloids, crambescins and crambescidins, originating from the marine sponge *Crambe crambe* (Crambeidae). Cramb816 (**21**) has been identified as a partial blocker of the voltage-gated calcium channels (CaV) and voltage-gated sodium channels (NaV) in neurons. This action reduces neurotransmitter release and synaptic transmission in the central nervous system, which may influence cell growth (Martin et al. 2013). Furthermore, the marine sponge species *Haliclona tubifera* George & Wilson (Chalinidae) (**22**), abundant along the Brazilian coastline, has shown significant cytotoxic effects and the ability to inhibit the production of peroxyl radicals (Biegelmeyer et al. 2015). Numerous sponge-derived compounds exhibit varying degrees of inhibition, ranging from moderate to potent, against the growth of human glioma cells (Neupane et al. 2019).

Ascidians, marine invertebrates with high bioactivity, have found extensive applications in cancer treatment (Watters 2018). Meridianins represent a group of marine natural compounds that exhibit various levels of kinase inhibition extracted from ascidian *Aplidium meridianum*, Sluiter (Polyclinidae). Meriolin 15 (**23**) has antiproliferative and pro-apoptotic activities against glioma and intratumoural endothelial cells. Meriolin 15 demonstrates IC_50_ values of 46 nM for SW1088 cells and 5.1 nM for U87 cells, respectively. Its impact includes inhibition of cell proliferation, reduction of undifferentiated cells and vascular components, and induction of cell death, in part through a caspase-3-dependent apoptotic pathway (Jarry et al. 2014).

Vitilevuamide, a bicyclic 13-amino acid peptide, was isolated from two marine ascidians, *Didemnum cuculiferum* Sluiter (Didemnidae) and *Polysyncraton lithostrotum* Brewin (Didemnidae). This peptide inhibited purified tubulin polymerization and reduced cell viability *in vitro*, with IC_50_ values of approximately 2 μM (Edler et al. 2002). Additionally, trichobamide A (**24**), isolated from the ascidian-derived fungus *Trichobotrys effuse* 4729, significantly inhibited proliferation in two glioma cell lines, U251 and SNB19 (Chen S et al. 2019). These findings lay a promising foundation for developing ocean-derived compounds into effective targeted drugs.

The jellyfish extract has shown inhibitory effects on glioma cell growth. The crude venom extract of the Mediterranean jellyfish *Pelagia noctiluca* (Pelagiidae) was fractionated into four fractions (F1, F2, F3 and F4) using Sephadex G-75 chromatography. U87 cells cultured with 10 μg/mL of venom or its fractions (F1–F4) for specified periods were assessed for cell proliferation activity using the MTT assay. Among these, fraction F3 showed a slight inhibitory effect on glioma cell growth, reducing it by approximately 15%. F4 did not show an effect, while F1 and F2 demonstrated greater effectiveness. These fractions could inhibit U87 cell viability in a time- and dose-dependent manner, with IC_50_ values of 125 and 179 μg/mL (Ayed et al. 2012). The bioactive sulphated saponins (**25**) of the sea cucumber *Holothuria moebii* Ludwig (Holothuriidae) had an activity that suppressed the proliferation of four different glioma cells with IC_50_ values ranging from 0.99 to 8.64 µM (Yu et al. 2015).

### Natural anti-glioma products from marine-associated plants

**Figure 3.**
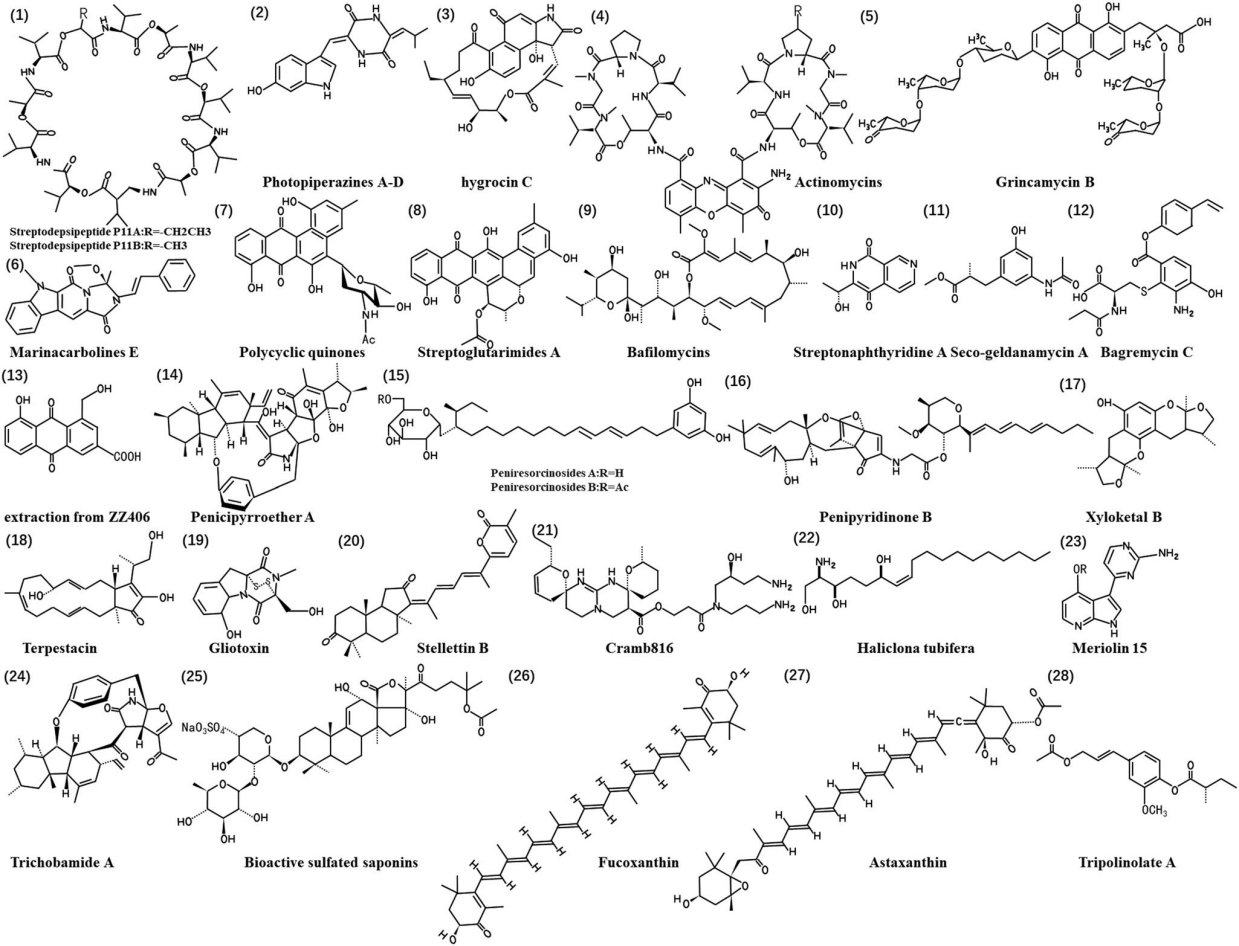
Chemical structures of the anti-glioma marine natural products (**1**-**28**).

Marine plants have long served as vital sources of drugs to combat human and animal diseases. Polyphenols derived from marine halophytes have shown activity in inhibiting glioma cell proliferation through a caspase-dependent pathway mediated by reactive oxygen species (ROS). These polyphenols have been found to impact proteins involved in the MAPK signalling pathway, such as the MMK3, p53, p70 S6 kinase and RSK1 (Murugesan et al. 2023). Two notable xanthophylls, astaxanthin (**27**) and fucoxanthin (**26**), commonly found in *Haematococcus pluvialis* (Haematococcaceae), *Phaffia rhodozyma* (Mrakiaceae), Phaeophyceae and Bacillariophyta, demonstrated toxicity at doses greater than 200 µM and 8 µM, respectively. Astaxanthin and fucoxanthin were found to limit considerable DNA damage (about 18 times) caused by 3 mJ/cm^2^ UV radiation in C6 cells. UV radiation led to down-regulation of the MRN complex, checkpoint kinase (Chk)1/2 activation, heterochromatin protein 1 (HP1)γ and mortalin, and up-regulation of the DNA damage markers 53BP1 and phosphorylated ATR. However, cells pretreated with astaxanthin and fucoxanthin showed a significant recovery in MRE11 expression. Non-toxic doses of these compounds prevented protein aggregation and protein misfolding and induced differentiation in glioma cells (Afzal et al. 2019). Furthermore, the PI3K inhibitor LY-294002 and fucoxanthin were found to modulate several common pathways, including inhibition of cell proliferation, DNA damage, DNA replication and cell cycle pathways (Pruteanu et al. 2020). Tripolinolate A (**28**), isolated from a *Tripolium pannonicum* (Jacq.) Dobrocz. (Asteraceae) halophyte, inhibited the proliferation of different glioma cells with IC_50_ values of 7.97–14.02 µM and had a significant inhibitory effect on the glioma growth in U87MG xenograft nude mice (Chai et al. 2018). In conclusion, a growing body of research demonstrates that marine-derived substances inhibit glioma cell proliferation and exhibit antitumour effects. Figure 3 shows the chemical structures of natural products (**1**-**28**).

## The marine-derived natural products that affect the migration and invasion of glioma cell

Migration and invasion are critical cellular mechanisms involved in tumour metastasis. Astaxanthin, a natural carotenoid derivative found in microalgae and marine organisms, has been investigated for its impact on glioblastoma cells (A172). MTT assay results indicate that astaxanthin is not toxic to A172 cells. In wound scratch and Boyden chamber assays, astaxanthin inhibited *in vitro* migration and invasion of A172 cells in a time- and concentration-dependent manner. These effects were associated with regulating matrix metalloproteinases (MMPs), with significant reductions in MMP-2 and MMP-9 observed after astaxanthin treatments. This suggests that astaxanthin may protect against glioblastoma metastasis (Siangcham et al. 2020).

Coibamide A (**29**), isolated from a marine cyanobacterial assemblage collected within the Coiba National Park in Panama (Medina et al. 2008), has shown significant anti-glioblastoma activity. U87-MG cells exhibited a high invasive capacity at the beginning of the assay, reaching a plateau in cell index after 12–15 h, while SF-295 cells invaded slowly and continuously for 48 h. Coibamide A exhibited inhibitory effects on cell migration and demonstrated antiangiogenic properties. It effectively suppressed tumour growth in a subcutaneous mouse model of glioblastoma. In summary, Coibamide A showed potent antitumour activity *in vitro* and *in vivo* (Serrill et al. 2016).

Motuporamines (**30**), extracted from marine sponges, have been shown to inhibit invasion in U-87 and U-251 glioma cells (Lin et al. 2001). Another marine compound, xyloketal B, is derived from the mangrove fungus *Xylaria sp*. 2508 and has been found to exert antiproliferative and migration effects on U251 cells by modulating the PI3K/Akt and MEK/ERK signalling pathways (Chen WL et al. 2015). In summary, compounds sourced from the marine environment inhibit the migration and invasion of glioma cells, ultimately contributing to their antitumour effects.

## The marine-derived natural products that affect the metabolism of glioma cells

Cell metabolism encompasses a series of organized chemical reactions within cells that are crucial to maintaining life. The three key metabolic pathways are glycolysis, the tricarboxylic acid cycle (TCA) and oxidative phosphorylation (Martínez-Reyes and Chandel 2021). Regulation of tumour cell metabolism significantly influences glioma cell survival (Guo D et al. 2022). Studies have indicated that certain marine-derived compounds also modulate glioma cell metabolism.

### The marine-derived natural products that affect the ATP consumption of the glioma cell

ATP is the energy exchange factor that bridges anabolism and catabolism, which plays a crucial role in motile contraction, phosphorylations and active transport (Bonora et al. 2012). Mandelalides (A to L) (**31**), originally isolated from a newly discovered species of *Lissoclinum tunicate* Monniot F. & Monniot C (Didemnidae) in and around Algoa Bay in the Eastern Cape of South Africa, constitute a family of polyketide macrolactones. Treatment with mandelalide A induces time- and concentration-dependent increases in AMPKα (Thr172) and ACC (Ser79) phosphorylation states compared to control U87MG cells treated with drug-loaded (0.1% DMSO) alone. This suggests that mandelalide induces an indirect AMPK activation mode to maintain cellular ATP and nucleotide balance in response to energy stress. As a result, cytotoxic mandelalides represent a class of potentially valuable inhibitors of ATP synthase (Mattos et al. 2022), which affect cell metabolism.

### The marine-derived natural products that affect the lipid metabolism of the glioma cell

Lipids play essential biological roles in the body, including energy storage and acting as signalling molecules and structural components of membranes (Martin-Perez et al. 2022). Regulation of lipid metabolism can affect tumour cell viability. Aplysinellamides A–C (**32**), isolated from an Australian *Aplysinella* sp. (Aplysinellidae) marine sponge, exhibit notable effects. Among these compounds, Aplysamine-1 significantly modulates apolipoprotein E (ApoE) and doubles its secretion from human CCF-STTG1 astrocytoma cells at a concentration of 30 μM. ApoE is a major apolipoprotein in the central nervous system and plays a central role in cholesterol transport.

**Figure 4.**
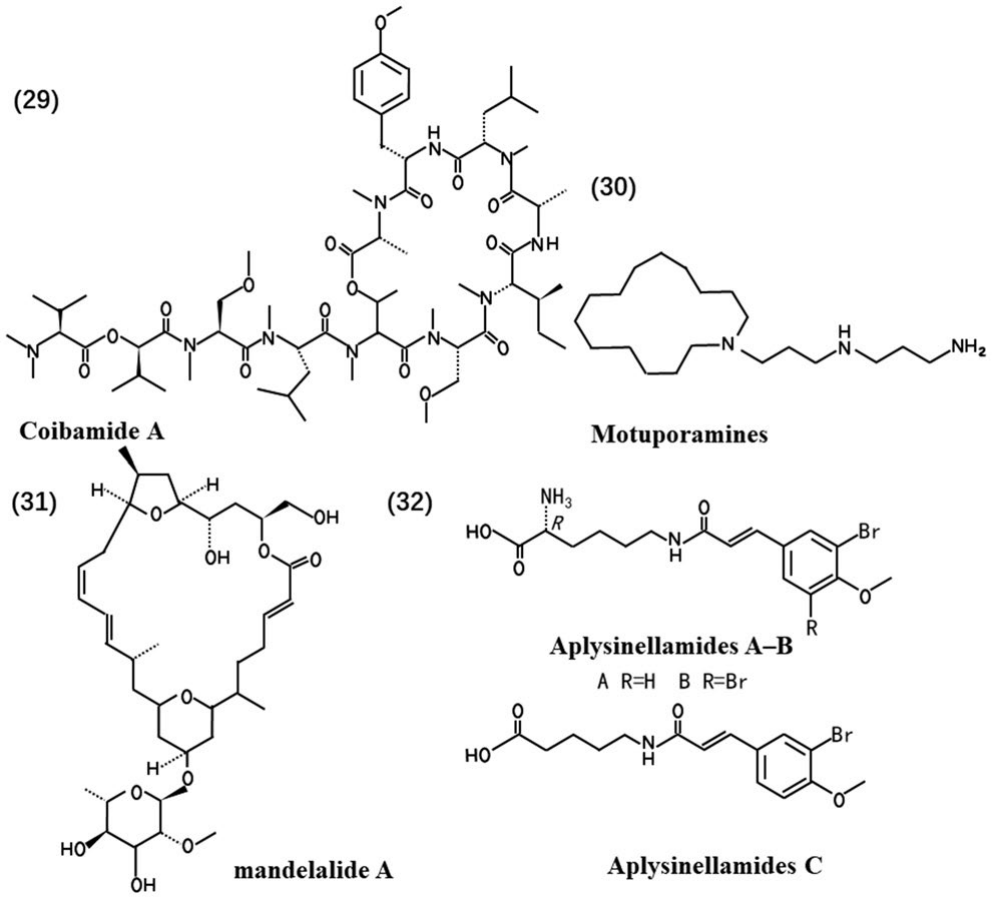
Chemical structures of the anti-glioma marine natural products (**29**-**32**).

Aplysinellamide A restricts the binding of ApoE to lipids, such as cholesterol, thus regulating lipid metabolism (Tian LW et al. 2014). Maitotoxin, a potent marine toxin, induces the accumulation of inositol 1,4,5-trisphosphate in a time-dependent manner. Maitotoxin stimulates phosphoinositide hydrolysis in an extracellular Ca^2+^-dependent manner but not intracellular Ca^2+^ in 1321N1 human astrocytoma cells (Nakahata et al. 1999). Phosphoinositides, lipid signalling molecules derived from phosphatidylinositol, act as master regulators of cell signalling (Posor et al. 2022). Maitotoxin regulates the processes of signalling and lipid transport. These findings collectively demonstrate that marine-derived compounds can influence glioma cell lipid metabolism, thus exerting antitumour effects. Streptodepsipeptide P11A and P11B induced glioma cell death with down-regulation of key metabolic regulators, including hexokinase 2 (HK2), phosphofructokinase-2/fructose-2,6-bisphosphatase 3 (PFKFB3), PKM2, glutaminase (GLS) and fatty acid synthase (FASN), which govern glycolysis, glutaminolysis and lipogenesis (Ye et al. 2017). Therefore, marine-derived natural products affect the lipid metabolism of the glioma cell. Figure 4 shows the chemical structures of natural products (**29**-**32**).

## The marine-derived natural products that affect the death of glioma cells

**Figure 5.**
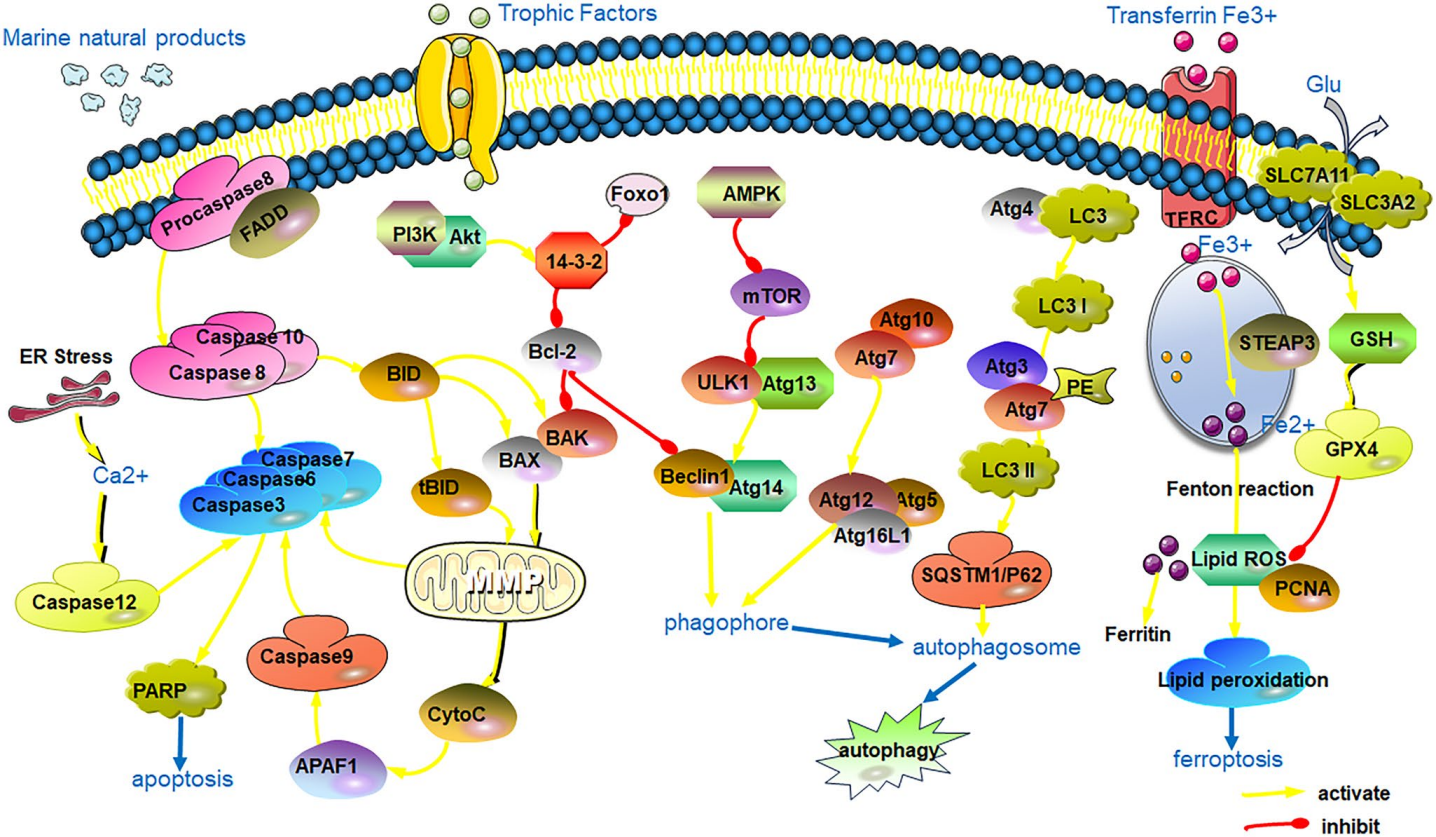
Mechanisms of glioma cell death induced by marine-derived compounds.

The discovery of regulated cell death processes has significantly advanced cancer treatment. Two main types of cell death are recognized: regulatory cell death (RCD) and accidental cell death (ACD) (Galluzzi et al. 2018). ACD is an uncontrolled process resulting from unintentional injury, where damage stimulation exceeds the cell’s regulatory capacity, leading to cell death. RCD, also known as programmed cell death (PCD), involves the spontaneous and orderly death of cells controlled by genes to maintain the stability of the internal environment. Various known types of RCD include autophagy-dependent cell death, apoptosis, necroptosis, pyroptosis, ferroptosis, parthanatos, entosis, NETosis, lysosome-dependent cell death (LCD), alkaliptosis and oxeiptosis (Christgen et al. 2022). Marine-derived compounds have shown the ability to induce apoptosis, autophagy and ferroptosis in glioma cells, thus limiting tumour growth. This opens possibilities for rationally developing marine-derived compounds as potential antitumour drugs (Figure 5).

### The marine-derived natural products that affect glioma cell apoptosis

Apoptosis is a controlled cell death independent, organized and orchestrated by multiple genes. It can occur through one of three main pathways, including an endogenous pathway that involves the release of Bcl-2-mediated mitochondrial cytochrome C, an exogenous pathway mediated by the expression of the death receptor ligand, and the dependent cytotoxic T lymphocyte/natural killer cell (CTL/NK) pathway (Wu et al. 2023). Compounds such as *Polymastia janeirensis* (da Frota et al. 2009), candidaspongiolide (**33**) (Trisciuoglio et al. 2008) and eupalmerin acetate (**34**) (Iwamaru et al. 2007), sourced from marine sponges or Caribbean gorgonian octocorals, induce caspase-dependent apoptosis through the endogenous pathway in human glioma cell lines.

Crude venom from the Persian Gulf Snail (Conus textile) has been found to combat U87MG human glioma cells by activating intrinsic apoptosis pathways (Salimi et al. 2021). Additionally, α-carboline derivatives TJY-16 (**35**) (Huang et al. 2016) and SZ-685C (**36**) (Xie G et al. 2010), isolated from marine animals or marine-derived mangrove endophytic fungi, induce arrest and apoptosis of the G2/M cell cycle in glioma cells through extrinsic and intrinsic apoptotic pathways. Marine-derived compounds can also induce glioma cell apoptosis through alternative pathways. For example, 1′-deoxyrhodoptilometrin and (*S*)-(−)-rhodoptilometrin (**37**), isolated from the marine echinoderm *Comanthus* sp. (Aplysinellidae), induce apoptotic and necrotic cell death by inhibiting the EGF receptor/MAPK pathway (Wätjen et al. 2017). Furthermore, 1,2,3,4-tetrahydroisoquinoline alkaloids from Thai marine invertebrates induce apoptosis in U373MG cells through the ErbB (EGFR) signalling pathway, which involves focal adhesion kinase (FAK)/PTK2, AKT3 and glycogen synthase kinase 3 Beta (GSK3B) (Tabunoki et al. 2012). Turbinamide (**38**), isolated from the marine ascidian *Sydnium turbinatum* (Polyclinidae), induces apoptosis in C6 cells (Esposito et al. 2002). Saponins, natural glycosides, have significant anti-tumour activity by inducing apoptosis (Tian X et al. 2013). These diverse marine-derived compounds have the potential for therapeutic applications in glioma treatment.

### The marine-derived natural products that affect glioma cell autophagy

Autophagy is a process that directs damaged organelles to the lysosome through double-membraned vesicles for degradation (Mizushima and Komatsu 2011). Studies indicate that autophagy plays a bidirectional role in cancer (Kocaturk et al. 2019). Under certain conditions, excess or deregulated autophagy activity may lead to glioma cell death. Prodigiosin (**39**), a secondary metabolite isolated from marine *Vibrio* sp. (Vibrionaceae), has decreased cell viability, and can form neurospheres in glioblastoma cells. LC3 puncta formation and acridine orange staining in U87MG and GBM8401 human glioblastoma cells indicate that prodigiosin induces excessive levels of autophagy. These results suggest a potential mechanism through which prodigiosin may exert its anti-cancer effects. The autophagy inhibitor 3-methyladenine reverses prodigiosin-induced autophagic cell death.

The c-Jun N-terminal kinase (JNK) pathway and the AKT/mTOR pathway were implicated in prodigiosin-induced cell death, triggering autophagic cell death and apoptosis in glioblastoma cells (Cheng SY et al. 2018). Autophagy is regulated by autophagy-related genes such as UNC-51-like kinase 1 (ULK1), Beclin-1, light chain 3 (LC3), p62 and FoxO, each playing different roles in autophagy processes. ULK1 promotes autophagy by regulating its initiation (Mokarram et al. 2017). Similarly, aaptamine and its isoform isoaaptamine (**40**), alkaloids extracted from the tropical sponge *Aaptos* sp. (Suberitidae), have been shown to induce cell death in GBM 8401, U87 MG, U138 MG and T98G cells in a concentration- and time-dependent manner, with IC_50_ values ranging from 8 to 60 µM. Isoaaptamine specifically induces ROS overproduction, up-regulates autophagy-associated proteins p62 and LC3, and forms acid organelle vesicles related to autophagy, leading to autophagic cell death (Wen et al. 2023). These findings underscore the potential of marine-derived compounds to induce autophagic cell death in glioma cells, highlighting their importance in cancer therapy.

### The marine-derived natural products that affect glioma cell ferroptosis

Ferroptosis is an iron-dependent form of regulated cell death triggered by the toxic accumulation of lipid peroxides in cell membranes (Lei et al. 2022). Fucoxanthin (**41**), extracted from seaweed, exhibits antitumour effects by inhibiting the survival of GBM cells through the induction of ferroptosis. Fucoxanthin administration can block the transferrin receptor (TFRC) degradation and down-regulate the expression of proliferating cell nuclear antigen (PCNA), restricting cell growth *in vivo* and *in vitro* (Zhu et al. 2023). These findings highlight the significant inhibitory effects of marine-derived compounds on glioma by activating ferroptosis, providing a novel treatment option for patients with glioma.

## The marine-derived natural products that affect the immune cell response of glioma cells

The brain tumour microenvironment is highly immunosuppressive and differs from other malignancies due to the presence of glial, neural and immune cell populations (Andersen et al. 2021). Immunotherapy has shown effective therapeutic responses in various tumours, including brain tumours. Frondoside A (FA) (**42**), a triterpenoid glycoside isolated from sea cucumber, has emerged as a potential therapeutic agent. It suppresses the expression of MYC and its downstream gene targets, including cyclin-dependent kinases and other oncogenic regulators. FA induces brain tumour cell death with an IC_50_ value of 0.37 μM. Administration of FA potently induces cytotoxicity in tumour xenografts, significantly extending the survival of tumour-bearing animals.

**Figure 6.**
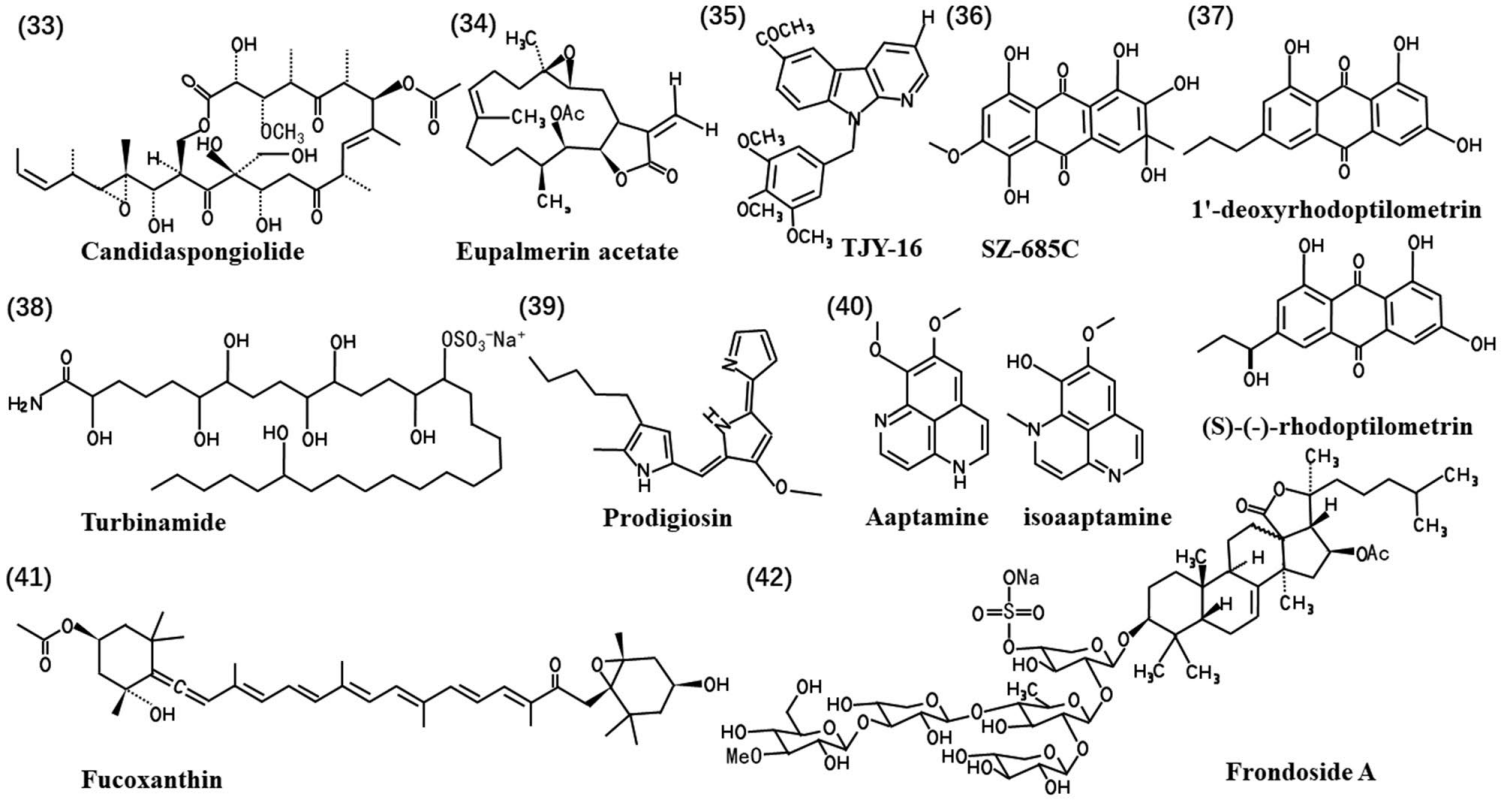
Chemical structures of the anti-glioma marine natural products (**33**-**42**).

FA-treated xenografts exhibited a higher abundance of Iba1 microglia/macrophages and CD8 cytotoxic T lymphocytes (CTLs) in the periphery than mock-treated tumours. This region also contained more perforin-labelled cells, a key component of cytotoxic granules released by active CTLs and natural killer cells. These findings suggest a potential role for FA in modulating tumour-associated immune responses (Xue et al. 2021). Figure 6 shows the chemical structures of natural products (**33**-**42**).

## The marine-derived natural products that affect the stemness of glioma cells

Glioblastoma stem cells, a subpopulation of glioma cells, exhibit characteristics of stem cell. They can self-renew, proliferate and differentiate into various cell types, which underlies cellular heterogeneity and plays a pivotal role in the initiation, progression, resistance to treatment, and relapse of GBM (Sharifzad et al. 2019). Marine natural products have been shown to exhibit strong inhibitory effects on GSCs, providing a new avenue to explore novel drugs for GBM treatment. Grincamycin B inhibits the formation of GBM spheres and the expression of the GBM stem cell marker in a dose-dependent manner (Yao et al. 2020). The most common pathways involved in maintaining GSCs include the Notch signalling pathway, the Sonic hedgehog (SHH)/glioma-associated oncogene (GLI) signalling pathway and Wnt/β-catenin signalling pathway. However, these pathways are also shared by normal stem cells (Sharifzad et al. 2019). The Notch signalling pathway maintains the stemness of GSCs and promotes cell migration. The SHH/GLI signalling pathway and the Wnt/β-catenin signalling pathway are crucial for CNS self-renewal, differentiation and neural stem cell development. The NF-κB pathway regulates the self-renewal capacity of CSCs, the JAK-STAT pathway, the tumour growth factor (TGF)/SMAD pathway, the PI3K/AKT/mTOR pathway and the EGFR pathways (Yang et al. 2020; Nasrolahi et al. 2023). Grincamycin B decreased PI3K/AKT activation and targeted RHOA in GSCs. RHOA can affect cancer stem cell (CSC) phenotypes through WNT/β-catenin signalling. GCN B is a potential RHOA and PI3K/AKT pathway inhibitor, which might be a dual inhibitor to treat GBM (Yao et al. 2020). Meanwhile, the principal pathway in which marine-derived compounds regulate CSCs has not been explored.

## Discussion and conclusions

This review summarizes the sources, structures, *in vitro* pharmacological activities and mechanisms of marine-derived compounds to explore their potential in treating GBM. Compounds of marine origin and their derivatives exhibit inhibitory effects on cell proliferation, migration and invasion. They also regulate cell metabolism and stem, induce cell death in glioma, and modulate the immune response in GBM (Table 1).

**Table 1.** Activities of marine sources and their derivatives against glioma cells.

Name	Source	Cell line	IC_50_	Bioactivity	*In vivo* test	Ref.
Streptodepsipeptide P11A	*Streptomyces* sp. P11-23B	U87-MG, U251	0.3–0.4 mΜ, 0.1–1.4 μM	Induce apoptosis, down-regulate key metabolic regulators		Ye et al. (2017)
Photopiperazines A–D	*Actinomycetes*	U87	0.12 μM	Cytotoxic		Kim et al. (2019)
Hygrocin C	*Actinomycetes*	U87MG, U251	0.16 μM, 0.35 μM	Antiproliferation		Yi, Newaz, et al. (2022)
Actinomycins D, V, X0β	*Actinomycetes*	U251, SHG44, C6	1.01–10.06 nM, 0.42–1.80 nM, 3.26–25.18 nM	Antiproliferation		Zhang X et al. (2016)
Grincamycin B	*Actinomycetes*	U251, 091214	0.5–2 μM, 0.75–3 μM	Antiproliferation, targeting ROHA and PI3K/AKT		Yao et al. (2020)
Marinacarbolines E	*Actinomycetes* sp. ZZ1866	U87MG	2.3–8.9 μM	Cytotoxic		Qin et al. (2020)
Polycyclic quinones	*Streptomyces* sp. 182SMLY	U87MG, U251, SHG44, C6	0.5–7.3 μM	Antiprolifearion, induce apotosis		Liang et al. (2016)
Streptoglutarimides A–J	*Streptomyces*	U87MG, U251	1.5–3.8 μM, 0.05–0.22 μM	Antiproliferation		Zhang D et al. (2019)
Bafilomycins	*Streptomyces*	U87-MG, U251, SHG-44	0.35–2.95 µM	Antiproliferation		Zhang X et al. (2017) and Zhang Z et al. (2017)
Streptonaphthyridine A	*Streptomyces* sp. SY2111	U87MG, U251	7.9 ± 1.3 µM, 13.4 ± 2.7 µM	Antiproliferation		Qin et al. (2023)
Seco-geldanamycin A	*Streptomyces* sp. ZZ1944	U251, U87MG		Antiproliferation		Yi, Lian, et al. (2022)
Bagremycin C	*Streptomyces* sp. Q22	U251, U87MG, SHG44, C6	2.2–6.4 μM	Cytotoxic, G0/G1 cycle arrest		Chen L et al. (2017)
Extraction from ZZ406	*Streptomyces* sp. ZZ406		4.7–8.1 μM	Cytotoxic		Chen M et al. (2018)
Peptide	Bacterium *Brevibacillus* sp. S-1	U251		Induce apoptosis		Zheng et al. (2014)
Penicipyrroether A	*Penicillium* sp. ZZ380	U87MG, U251	1.64–5.50 μM	Antiproliferation		Song et al. (2019)
Peniresorcinosides A, B	*Penicillium* sp. ZZ1750	U87MG, U251	4.0 and 5.6 µM, 14.1 and 9.8 µM	Antiproliferation		Yong et al. (2021)
Penipyridinone B	*Penicillium* sp. ZZ1750	U87MG, U251	2.45 and 11.40 µM	Antiproliferation, induce apoptosis		Yong et al. (2022)
Xyloketal B	Mangrove fungus *Xylaria* sp.	U251	287.1 ± 1.0 μM	Anti-proliferation and migration effects		Chen WL et al. (2015)
Terpestacin	Coral endophytic fungus *Arthrinium*	U87MG	2.8–6.9 μM	Cytotoxic		Liao et al. (2023)
Gliotoxin	Fungal metabolite	U87	0.54 mM	Cytotoxic		Tang W et al. (2020)
Stellettin B	Marine sponge	SF295	0.01 μM	Antiproliferation, induce apoptosis		Tang SA et al. (2014) and Peng et al. (2023)
Cramb816	Marine sponge	NG 10815	1.2 μM	Decrease the neurotransmitter release and synaptic transmission		Martin et al. (2013)
Haliclona tubifera	Marine sponge	U87	12.47 μg/mL	Antioxidant capacity, affect cell metabolism		Biegelmeyer et al. (2015)
Meriolin 15	Marine ascidian	SW1088, U87	46 nM, 5.1 nM	Inhibit cell proliferation	U87MG xenograft mice:36.6% inhibition of tumour growth at 2 mg/kg/day for 2 days	Jarry et al. (2014)
Vitilevuamide	Marine ascidian		2 μM	Inhibit polymerization of purified tubulin		Edler et al. (2002)
Trichobamide A	Marine ascidian-derived fungus	U251, SNB19		Antiproliferation, induce apoptosis		Chen S et al. (2019)
Fractions (F1, F2)	Mediterranean jellyfish	U87	125,179 μg/mL	Antiproliferation		Ayed et al. (2012)
Bioactive sulfated saponins	Sea cucumber	U251, U87MG, SHG44, C6	0.99–8.64 µM	Antiproliferation, induce apoptosis		Yu et al. (2015)
Polyphenols	Marine halophyte	LN229	11.95 μg/mL	Inhibit cell proliferation		Murugesan et al. (2023)
Astaxanthin	*Haematococcus pluvialis, Phaffia rhodozyma*	C6, A172	150 µM	Inhibit cell migration and invasion		Afzal et al. (2019)
Fucoxanthin	*Phaeophyceae, Bacillariophyta*			Inhibit cell migration and invasion		Siangcham et al. (2020)
Tripolinolate A	*Tripolium pannonicum*	U87MG	7.97–14.02 µM	Inhibit cell proliferation	U87MG xenograft mice: 55.2% inhibition of tumour growth at 120 mg/kg/day for 21 days	Chai et al. (2018)
Coibamide A	Cyanobacterial	U87-MG, SF-295	1.4 µM	Inhibit cell migration and invasion	U87MG xenograft mice: 80% inhibition of tumour growth at 0.3 µg/kg/day for 21 days	Serrill et al. (2016)
Mandelalide A	*Lissoclinum*	U87MG	0.03–300 nM	Inhibit mitochondrial ATP synthase		Mattos et al. (2022)
Aplysinellamides A–C	Marine sponge	CCF-STTG1 cells		Regulate lipid metabolism		Tian et al. (2014)
Maitotoxin	Marine toxin	1321N1 cell		Elicit inositol 1,4,5-trisphosphate accumulation		Nakahata et al. (1999)
Polymastia janeirensis	Marine sponge	U138	<30 μg/mL	Induce apoptosis		da Frota et al. (2009)
Candidaspongiolide	Octocorals	U251		Induce apoptosis		Trisciuoglio et al. (2008)
Eupalmerin acetate	Marine sponge, Caribbean octocorals	U87-MG, U373-MG	5.1 μM, 6.9 μM	Induce caspase-dependent apoptosis	U87MG xenograft mice:80% inhibition of tumour growth at 50 mg/kg/day for 19 day	Iwamaru et al. (2007)
Conus textile	Persian Gulf Snail Crude Venom	U87MG	5–10 µM	Induce caspase-dependent apoptosis		Salimi et al. (2021)
TJY-16	Marine animals	C6, U87, T98G, U251	0.05–0.088 µM	Induce apoptosis	U87 xenograft mice:40% inhibition of tumour growth at 24 mg/kg/day for 10 days	Huang et al. (2016)
SZ-685C	Mangrove endophytic fungi	LN-444	7.8 µM	Induce G2/M cell cycle arrest and apoptosis	MDA-MB-435 xenograft mice: 61% inhibition of tumour growth at 50 mg/kg/day for 20 days	Xie G et al. (2010)
1′-Deoxyrhodoptilometrin and (S)-(−)-rhodoptilometrin	Marine echinoderm	C6	23.2 µM, 30 µM	Induce apoptotic and necrotic cell death		Wätjen et al. (2017)
1,2,3,4-Tetrahydroisoquinoline alkaloids	Marine invertebrates	U373MG	1.70–4.83 nM	Induced apoptosis		Tabunoki et al. (2012)
Turbinamide	Marine ascidia	C6	0.01–100 µM	Induced apoptosis		Esposito et al. (2002)
Prodigiosin	Marine *Vibrio* sp.	U87MG, GBM8401	7.36 µM, 12.29 µM	Induced autophagic cell death and apoptosis		Cheng et al. (2018)
Aaptamine isoaaptamine	Marine sponge	GBM8401, U87MG, U138MG, T98G	45–60 µM, 8–60 µM	Induced autophagy death		Wen et al. (2023)
Fucoxanthin	Seaweeds	U87MG	20 μM	Induced ferroptosis		Zhu et al. (2023)
Frondoside A	Sea cucumber	U87MG, iMB cell	0.37 μM	Modulate tumour-associated immune responses	iMB xenograft mice: 95% inhibition of tumour growth at 4 ng/kg/day for 20 days	Xue et al. (2021)

Glioma is a highly malignant cancer in the brain. First-line therapy for GBM is surgery followed by radiation and the alkylating chemotherapeutic agent TMZ (Schaff and Mellinghoff 2023). TMZ was approved by the FDA in 2005 based on a marginal increase (∼2 months) in OS (Karve et al. 2024). TMZ is converted to an alkylating methyldiazonium cation, leading to cell death by breaking the DNA double-strand. However, TMZ treatment faces challenges due to factors such as the BBB, tumour heterogeneity, GSCs, drug efflux pumps, DNA damage repair mechanisms, and high expression of the MGMT gene, all of which contribute to resistance to TMZ therapy (Angom et al. 2023; Fang 2024). Compounds derived from marine sources exhibit various origins and mechanisms, inhibiting glioma cell growth and migration through various means, such as inducing multiple modes of cell death and regulating immune cell activity. In recent years, immunotherapy and targeted therapy have been extensively studied and clinically tested in various tumours, demonstrating specific therapeutic effects. Both *in vitro* and *in vivo* experiments have shown that many compounds exhibit lower IC_50_ values compared to TMZ treatment. Furthermore, combining these compounds with TMZ results in a more effective inhibition than using TMZ alone. Therefore, marine-derived compounds provide a multi-modal approach, combining different therapies or therapeutic agents with distinct molecular mechanisms, to enhance the efficacy of glioma cell eradication.

Recent studies have highlighted that in addition to TMZ, the standard medication for glioma treatment, numerous natural compounds show promise as treatment options. These include betulinic acid (Fernandes et al. 2024), perillyl alcohol (Alharbi et al. 2024), matrine (Qiu et al. 2024), 9′-lithospermic acid methyl ester (Tzitiridou et al. 2024), cannabinoid compounds (Pires et al. 2024), eugenol (Padhy et al. 2022), curcumin and flavonoids (Persano et al. 2022). For example, magnolol, a hydroxylated biphenyl compound isolated from the bark of *Magnolia officinalis* Rehder & Wilson (Magnoliaceae), suppresses cell proliferation and induces cell cycle arrest and apoptosis at a concentration of 50 µM by inhibiting DNA synthesis in glioma cells (Chen and Lee 2013). This concentration is significantly higher than the IC_50_ value for compounds of marine origin. Furthermore, magnolol induces cytotoxic autophagy in glioma by inhibiting the PI3K/AKT/mTOR signalling pathway (Cheng YC et al. 2018). It can potentiate the apoptotic effects of TMZ by inhibiting MGMT activation mediated by the NF-κB pathway (Kundu et al. 2023).

Although marine-derived compounds offer unique advantages in glioma treatment, such as their ability to cross the BBB and influence cell metabolism, immune cell response, and the stemness of glioma cells, there are still significant gaps in our understanding. The mechanisms underlying resistance to TMZ, a key challenge in glioma treatment, require further investigation. Most studies are limited to reporting IC_50_ values and using bioinformatics for predictive analysis. Comprehensive in-depth mechanistic studies and *in vivo* experiments are crucial in advancing these marine compounds into viable therapeutic options for GBM.

In conclusion, exploring ocean-derived drugs for treating GBM is still in its early stages and requires more preclinical and clinical research. This review provides a comprehensive overview of marine natural products with potential applications in GBM treatment, facilitating ongoing scientific research and serving as a valuable resource for researchers selecting drug candidates for further exploration.
